# A speed-oriented phenotype is beneficial for 1500 m rowing at the Los Angeles 2028 Olympic Games

**DOI:** 10.1007/s00421-026-06250-5

**Published:** 2026-05-16

**Authors:** Erik Baumann, Emma M. C. van der Vorst, Mathijs J. Hofmijster

**Affiliations:** 1 Faculty of Behavioural and Movement Sciences, Vrije Universiteit, Amsterdam, The Netherlands; 2 Amsterdam Institute of Sport Science, Amsterdam, The Netherlands; 3 Amsterdam Movement Sciences – Sports, Amsterdam, The Netherlands

**Keywords:** Locomotor profiling, Force-velocity profiling, Rowing performance, Endurance sport, Exercise physiology

## Abstract

**Purpose:**

The shorter rowing course at the Los Angeles 2028 Olympics, which is 1500 m instead of 2000 m, potentially alters the physiological demands of racing. This study aims to elucidate whether the shortened distance favours rowers with a more speed-oriented phenotype, using two non-invasive profiling approaches: locomotor profiling and force-velocity (F-v) profiling.

**Methods:**

Nine nationally competitive male rowers completed 2000 m and 1500 m rowing ergometer time trials. Peak power tests and graded exercise tests were used to determine the athletes’ locomotor profiles expressed as Power Reserve Ratio (PRR) and loaded back squat testing was performed to establish individual F-v profiles. Relative performance was expressed as the ratio between mean power output of the 1500 m and 2000 m time trials (MPO_ratio_).

**Results:**

PRR showed a strong positive correlation with MPO_ratio_ (*r* = 0.61), indicating that athletes with a more speed-oriented locomotor profile performed relatively better over the shorter distance. F-v slope demonstrated a moderate positive correlation with MPO_ratio_ (*r* = 0.36), which became very strong after excluding an outlier (*r* = 0.88).

**Conclusion:**

Together, these findings support the notion that the shortened Olympic rowing distance places greater emphasis on speed -ability, a challenge that suits athletes leaning towards a more speed-oriented phenotype. While aerobic capacity remains essential in 1500 m rowing, subtle adjustments in athlete selection and training toward increased anaerobic and explosive qualities may optimize performance at the Los Angeles 2028 Olympics.

## Introduction

Since the Stockholm 1912 games the Olympic rowing distance has been standardized to 2000 m. Unsurprisingly, most rowing-specific physiology research has been conducted on the 2000 m rowing distance (e.g., Ingham et al. 2002). The upcoming Los Angeles 2028 Olympic Games (LA28) propose an unfamiliar challenge, as the races will be held over 1500 m. Given the recency of the LA28 course announcement, there is a substantial research gap about the physiological differences between 1500 m- and the traditional 2000 m-rowing. Astridge et al. (2024) showed that, compared to a 2000 m ergometer trial, the 1500 m trial elicits 5.2% higher mean power outputs (MPO) and a 4.9% higher anaerobic contribution (22.2% rather than 17.3%). Boillet et al. (2025) used a mathematical modelling approach and a confirmation trial to simulate 2000 m and 1500 m single sculls races. They found that, for 1500 m compared to 2000 m, the importance of oxidative capacity (i.e., aerobic endurance) remained unchanged, but the importance of anaerobic capacity increased. They concluded that athlete training should not be drastically altered because the relative non-oxidative energy contribution remains low. Higgins and Hofmijster (2025) found that anaerobic contribution was approximately 10 per cent points greater in 4-minute time trials (estimated 1500 m time for Men’s Eight) compared to 6-minute 30 s time trials (estimated 2000 m time for Men’s single scull). While research thus far has addressed the description of expected differences between the two rowing distances, it has not yet examined various aspects of the underlying physiology.

The finding that anaerobic contribution increases notably in the shortened race duration suggests that the optimal rower phenotype may differ between 1500 m and 2000 m races. An important aspect of athlete phenotype is fibre type distribution. Studies investigating muscle fibre type distribution in elite athletes are rare, since the gold-standard measurement tool, muscle biopsy, is highly invasive and thus generally unsuitable for high-performance athletes. Regardless, biopsies have been used in track-and-field, where it has been established that elite athletes in longer duration endurance events possess a higher percentage of slow twitch muscle fibres, both in number and cross sectional area, while the opposite, i.e., higher percentage of fast twitch fibres, is true for sprint and power athletes (Costill et al. 1976; Foster et al. 1978). For middle-distances the optimal makeup is not fully understood but seems to vary between athletes (van der Zwaard et al. 2018). For 2000 m rowing, it has been found that highly trained athletes have a large percentage (70–85%) of slow twitch fibres (Steinacker 1993). These high percentages correspond with the strong correlation between 2000 m rowing performances and maximal oxygen uptake capacity (V̇O_2_max), as well as power at V̇O_2_max (Ingham et al. 2002).

Research in cyclists has shown that there is an inverse relationship between normalized endurance and sprint power (van der Zwaard et al. 2018), this relationship is called “sprint-endurance continuum”. Athletes located at the endurance end of this continuum fare relatively better at long duration events, such as road cycling or (ultra-)marathon running. The opposite can be said about athletes on the sprint side of the continuum, who perform best at short duration events like the 100 m dash or sprint track cycling. 2000 m rowing requires both aerobic and anaerobic capacity (Volianitis and Secher 2009). The 500 m course reduction at LA28 causes a reduction in performance duration, resulting in a higher relative anaerobic energy production (Higgins and Hofmijster 2025), which should benefit athletes on the sprint-end of the continuum (hereafter termed “speed-oriented” athletes). Thus, our central hypothesis is that the shortened rowing course at LA28 is best suited to athletes with a more speed-oriented phenotype.

To investigate this hypothesis, we used two established proxies for muscle fibre type distribution determination: locomotor profiling (e.g., Sandford et al. 2019 a) and force-velocity slope profiling (e.g., Cormie et al. 2011; Giroux et al. 2017). Both are non-invasive profiling approaches that can be applied by rowing federations with relatively simple means. These approaches do not establish muscle fibre typology directly, rather they indirectly estimate an athlete’s physiological profile. This allows the categorization of athletes’ phenotypes in relation to their position on the sprint-endurance profile continuum.

Locomotor profiling, stems from an observation by Bundle et al. (2003) who found that, at intensities beyond V̇O_2_max, speed-duration relationships could be accurately predicted by two factors: maximum aerobic speed and maximum sprint speed. This principle can be applied to speed and power, depending on the endurance sport. The thereby created “anaerobic speed or power reserve” model predicts maximal speed- or power-duration relationships in short duration exercise up to five minutes (Bundle et al. 2003; Sandford et al. 2021). By using the model’s subcomponents, maximal peak power (MPP) and maximal aerobic power (MAP), the Power Reserve Ratio (PRR) can be obtained according to: *PRR = MPP/MAP*, which quantifies the locomotor profile. Relatively high peak powers compared with a reduced ability to sustain high power outputs yields a high PRR and are characteristic of athletes with a high percentage of fast twitch fibres, which is why locomotor profiles correlate with fibre type distribution (Sandford et al. 2021). A high PRR suggests a speed-oriented profile, while a low PRR suggests an endurance-oriented profile. Studies by Thron et al. (2024) and Sandford and Stellingwerff (2019 b) support this profiling approach in sprinting and middle distance running, as speed profile athletes (high PRR) fared relatively better at shorter distance disciplines. Results from Deguire et al. (2023) show that multi-event short track speed skaters with an endurance profile perform relatively better over longer distances compared to shorter distances. In middle-distance running, within a given race distance, locomotor profiles are variable (Sandford and Stellingwerff 2019 a). Athletes with different profiles can perform comparatively within their specialized distance; however, locomotor profiles may indicate which athletes would perform better at a shorter/longer distance. Thus, middle-distance athletes with a more speed-oriented locomotor profile would likely outperform athletes with a more endurance-oriented locomotor profile in shorter races. PRR profiling in rowing has recently been done for the first time in a study by Rappelt et al. (2025). They suggested that the optimal PRR for 1500 m performances might be higher than for 2000 m, but they did not investigate this hypothesis.

The force-velocity (F-v) relationship was first proposed by Hill (1938). It reflects the mechanical capacity of the neuromuscular system to generate force across varying contractile velocities. Like locomotor profiling, the underlying physiology reflects the muscle fibre type composition, with several studies demonstrating associations between fibre type distribution and F-v characteristics (Thorstensson et al. 1976; Cardona et al. 2016). There has been debate about the linearity of the F-v relationship but in maximum multi-joint movements against an external load, it has been proposed to be linear (Jaric 2015; Alcazar et al. 2019). Research using isolated muscle fibre bundles showed that the slope of the linear F-v profile of fast twitch fibres has a smaller magnitude (i.e., is less steep) than those of slow twitch fibres (Faulkner et al. 1982, 1986). These ex vivo studies are consistent with in vivo findings where athletes with different training backgrounds on the muscle-group level were compared: sprint- and power-trained athletes exhibit slopes with a smaller magnitude than rowers who have a high percentage of slow twitch fibres (Giroux et al. 2015, 2017; Larsson and Forsberg 1980). Thus, individuals with a more speed-oriented muscular profile tend to exhibit a smaller magnitude F-v slope compared to those with a more endurance-oriented muscular profile (Cormie et al. 2011). In our study, we used the back squat movement to determine individual F-v profiles since this movement primarily involves the quadricep muscles (Pavão et al. 2024), which is the dominant muscle group used in rowing (Guével et al. 2011).

In this study we aim to investigate whether locomotor profiling using the PRR, and F-v profiling using the F-v slope, are indicative of relative performance differences between 2000 m and 1500 m rowing. We hypothesize that PRR and F-v slope are both positively correlated to a relatively better performance over 1500 m rowing compared to the 2000 m distance. Ultimately, if confirmed, these hypotheses support the underlying notion that rowers leaning towards a more speed-oriented phenotype are better equipped to perform at the 500 m shorter rowing course at LA28.

## Methods

### Participants

Through social media outreach and personal contacts of the authors, a total of 17 athletes (10 male and 7 female) were recruited for this study. As an incentive, we provided a report with physiological metrics to each participant. For inclusion, athletes had to be at least nationally competitive (i.e., tier 3; McKay et al. 2022). Because five out of seven recruited female subjects were below tier 3, the female athletes were excluded from this paper. One male athlete reported pacing issues and sub-optimal performance during the 1500 m. As such, data from this participant were excluded from data analysis. The characteristics of the nine included athletes were (average ± SD): age: 25.0 ± 6.1 y, height: 190.2 ± 4.1 cm, weight: 84.5 ± 9.6 kg, V̇O_2_max: 4.67 ± 0.45 L×min^−1^.

All participants provided written informed consent prior to participating. The study was approved by the local ethical committee (VCWE-2024-096_R1).

## Procedures

### Outline

Participants performed 2000 m (TT_2000_) and 1500 m (TT_1500_) ergometer time trials at their usual testing environment before being invited to the exercise laboratory at the Vrije Universiteit Amsterdam. The laboratory testing was divided into two visits aimed at establishing F-v profiles and the PRR (with its subcomponents MPP and MAP). During visit one, MPP was established with an ergometer-based test, and F-v slopes were obtained through back squat testing. In visit two, participants performed a graded exercise test (GXT) on an ergometer, during which MAP and V̇O_2_max were determined.

Participants were asked to keep a normal diet, and refrain from alcohol, recreational drugs and strenuous exercise in the 24 h preceding every test and lab visit.

## Rowing ergometer time trials

The TT_2000_ and TT_1500_ ergometer time trials were performed in randomized order, within three to ten days of each other. Both trials were performed on a Concept2 RowErg (C2, Concept2 Inc., Morrisville, VT, USA) in a familiar setting at the participants’ own club. Drag factors were self-selected but were kept consistent across the time trials and the graded exercise test. Rate of perceived exertion (RPE) was collected after each time trial on a 0–10 adapted Borg scale (Foster et al. 2001). When RPE was 9 or 10, the tests were deemed maximal.

### Visit one

During the first laboratory visit the athlete’s height and weight were measured to the next 0.1 cm and 0.1 kg respectively. Weigh-ins were performed in a unisuit, which is standard equipment in rowing. Participants set up the C2 RowErg with their previously selected drag factor. Subsequently, testing began with a standardized warm-up protocol, which also served as a familiarization trial for the GXT performed during the second laboratory visit. To familiarize with the test and equipment, while wearing a gas exchange mask, the warm-up stage and the first three steps of the GXT were completed. Following the warm-up, the resistance was set to the maximum, and participants performed a practice trial of 10 maximal strokes. After five minutes of rest, participant performed their first ten stroke maximal test (10s_max_) trial. The procedure of five minutes rest and trial was repeated until three 10s_max_-trials were completed. To ensure optimal recovery, participants rested for 25 min before performing further testing.

For the squat test, a squat rack was set just below shoulder height and tape was placed on the floor, marking each participant’s starting stance to ensure consistency across trials. A stool was placed behind the participant and adjusted so that the knee angle would be 90° upon touching the stool. The warm-up consisted of 10 traditional back squats with a 20 kg barbell. Participants were instructed to perform the last three warm-up repetitions with maximal upward velocity to familiarize themselves with the movement required during the measured trials. Participants were then asked to perform the downward (eccentric) phase of the squat slowly until they touched the stool and the upward (concentric) phase with maximal velocity. Average velocity (v) was calculated for each repetition by measuring the displacement from minimum to maximum bar height using a linear position transducer (GymAware, Kinetic Performance Technologies, Canberra, Australia) and dividing this number by the time interval from initiation of the upward movement to the moment maximum height was achieved. Trials were discarded when maximum bar height deviated more than 5 cm from the height at the start of the trial. Average force (*F*) was determined by taking the average of the instantaneous force (*F*_*i*_) over said time-interval, according to *F*_*i*_ *= m·(a + g)*, with the acceleration of the bar (*a*), the gravitational acceleration 9.81 m·s^− 2^ (*g*), and the combined mass of the participant and the added load (*m*). Note that *a* is zero or close to zero on average as participants started and ended their movement with zero velocity. Each set consisted of two repetitions with a minimum of 3 min rest between sets. For each set the highest average upward velocity and force were used for further analysis. The first set was performed with the 20 kg barbell only. After each successful set, 15 kg of additional weights were added. Weight was increased until contractile velocities were between 0.3 and 0.5 m·s^− 2^. At least five data points are recommended for accurate F-v profiling (Morin and Samozino 2016). Therefore, all participants performed six or more sets to ensure a sufficient number of datapoints.

### Visit two

During the second and final visit, athletes performed the GXT protocol. The two visits were separated by a minimum of one hour and maximum of two weeks. Gas exchange data were acquired using a breathing gas analyser (COSMED Quark CPET, Rome, Italy). Breath-by-breath data were extracted and smoothed over 20 s using a customized MATLAB-script (The Math-Works Inc., Portola Valley, CA, USA). The test consisted of thirty seconds of measurements at rest, a three-minute warm-up stage at a very low prescribed power output, and finally a graded step protocol with fixed increments and a target power output for every minute. The protocol was designed to bring the athletes to their maximal exercise capacity and voluntary cessation of exercise due to exhaustion between 8 and 12 min. The starting values and increments depend on the participants’ MPO during the 2000 m ergometer test (MPO_2000_). The protocol is based on the finding that maximal minute power (which represents MAP in this study) is only ~ 6 W away from MPO_2000_ (Ingham et al. 2013). The warm-up was set at 3/13 of MPO_2000_ and the increments during the GXT were 1/13 of MPO_2000_. Within a 10 s range of TT_2000_ athletes have the same target values (i.e., 6:40.0–6:49.9 all performed the 6:45 protocol). Following Ingham et al. (2013), the test was terminated when participants reached volitional exhaustion or failed to be within 10% of their target wattage for five consecutive strokes. The test was deemed maximal if at least two of the following four criteria were fulfilled: respiratory quotient > 1.25, clear plateau in V̇O_2_, RPE ≥ 9, or heart rate achieved was ≥ 95% predicted maximal heart rate. Predicted maximal heart rate was calculated as *208 – 0.7 ∙ age* (Tanaka et al. 2001).

## Data processing

Mean power output of the self-reported TT_2000_ and TT_1500_ ergometer tests were calculated from the submitted completion times. For this the formula on the manufacturer’s website (Concept2) was used (Watts = 2.80/pace^3^, with pace in s/m). Drag factor does not affect this formula since the ergometer has a fixed power-to-speed relation.

For data analysis of the ergometer laboratory tests, stroke-for-stroke data were extracted with the Concept2 Logbook application. MPP was calculated as the mean value of the highest power output achieved during three consecutive strokes of all 10s_max_ trials. MAP was extracted as the highest average one-minute power during the GXT (Billat and Koralsztein 1996; Sandford et al. 2021). If an athlete terminated the GXT during an incomplete stage, the duration of this stage was divided by 60 and multiplied with the difference between ultimate and penultimate stage, then added to the penultimate stage’s MPO, as previously done by Bourdin et al. (2004). If participants stopped at the end of a GXT stage or failed to increase the power output at a subsequent stage switch, MAP was calculated as the average power output of that last completed stage. PRR was calculated as *MAP/MPP*.

To compute the individual F-v slopes, force was expressed as a function of velocity and fitted with a standard linear regression, following previous literature (Bobbert 2012; Iglesias-Soler et al. 2021; Rahmani et al. 2001). Consequently, the F-v slope was defined as the regression coefficient of the linear F-v relation, reflecting the rate at which average force decreases with increasing velocity. This is captured by *F(v) = [F-v slope]·v + F*_*0*_. Note that *F*_*0*_, the hypothetical force at zero velocity, was not used in further analysis.

### Statistical analysis

Statistical analyses were performed using JASP (JASP, Amsterdam, NL). For all variables, the mean and standard deviation (SD) were calculated. To examine the correlation between the PRR and relative rowing performance, and between the F-v slope and relative rowing performance, a two-tailed Pearson’s correlation (r) was used, after the verification of the required assumptions. Interpretation of Pearson’s correlation coefficients followed the thresholds recommended in sport science literature (Hopkins et al. 2009): trivial (< 0.1), small (0.1–0.3), moderate (< 0.3–0.5), strong (< 0.5–0.7), very strong (< 0.7–0.9), or almost perfect (< 0.9).

## Results

Average time trial completion time (in minutes: seconds) was 6:27.5 ± 14.1 s for 2000 m, and 4:45.3 ± 11.2 s for 1500 m, confirming that all athletes performed according to tier 3 (McKay et al. 2022). The calculated MPOs were 387.8 ± 43.1 W and 410.3 ± 48.3 W respectively. This constitutes an average MPO_ratio_ of 1.057 ± 0.024, indicating that power outputs during the 1500 m distance were on average 5.7% higher compared to 2000 m performance. The TT_2000_ completion times overlap with the on-water completion times in elite level small boat categories, as the completion times (6:27.5 ± 14.1 s) are very similar compared to the men’s pair medallists in the Tokyo and Paris Olympics and the two world championships in between (6:30.9 ± 14.1 s; World Rowing). This supports the validity of this paper’s findings for the LA28 Olympics. All variables are summarized in Table 1.

**Table 1:** Mean values, standard deviations (SD), and range of the main outcome variables of the study

Variables	Mean ± SD	Range
TT_2000_ (min: sec)	6:27.5 ± 14.1 s	6:06.2 – 6:50.1
MPO_2000_ (W)	387.8 ± 43.1	324.3 – 456.1
TT_1500_ (min: sec)	4:45.3 ± 11.2 s	4:28.1–5:05.2
MPO_1500_ (W)	410.3 ± 48.3	332.4 – 490.4
MPO_ratio_	1.057 ± 0.024	1.025 – 1.089
MAP (W)	424.7 ± 45.3	368 – 494
MPP (W)	835.9 ± 140.0	655 – 1008
PRR	1.97 ± 0.25	1.60 – 2.41
F-v slope (N·s·m⁻¹·kg⁻¹)	−17.36 ± 2.49	−14.77 to −22.42

*TT*_*2000*_ 2000 m ergometer time trial time, *MPO*_*2000*_  2000 m ergometer time trial mean power output, *TT*_*1500*_ 1500 m ergometer time trial time, *MPO*_*1500*_ 1500 m ergometer time trial mean power output, *MPO*_*ratio*_ ratio between 1500 m and 2000 m mean power outputs, *MAP* maximum aerobic power, *MPP * maximum peak power, *PRR* Power Reserve Ratio, *F-v slope * force-velocity slope

### Power reserve ratio

**Fig. 1:**
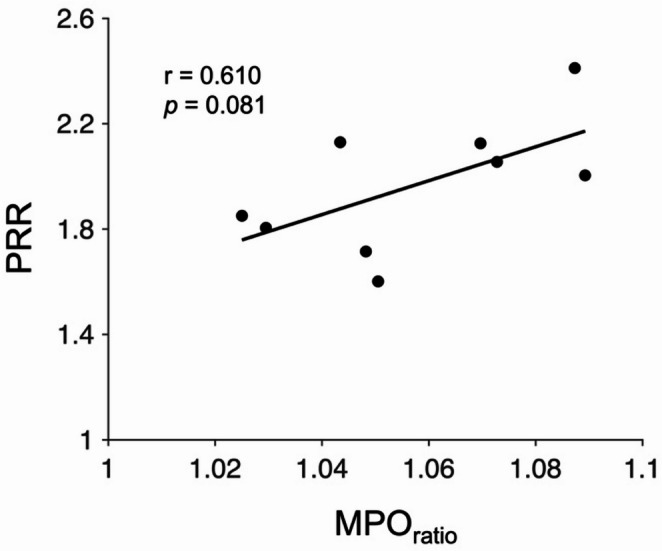
Correlation between Power Reserve Ratio (PRR) and the ratio between 1500 m Mean Power Output and 2000 m Mean Power Output (MPO_ratio_) (95% CI [−0.091, 0.907])

All participant’s GXTs were deemed maximal, as at least three of the outlined criteria were fulfilled. Average values of MAP and MPP were 424.7 ± 45.3 W and 835.9 ± 140.0 W, respectively. PRR ranged between 1.60 and 2.41 with an average of 1.97 (± 0.25). Figure 1 displays a scatterplot of the MPO_ratio_ and PRR relation. The correlation between MPO_ratio_ and PRR is strong and positive (*r* = 0.610, r^2^ = 0.372, *p* = 0.081, 95% CI [−0.091, 0.907]) indicating that individuals who perform relatively better at 1500 m compared to 2000 m generally have a higher PRR. Statistical significance level of *p* = < 0.05 was not reached. No outlier was identified in the correlation between PRR and MPO_ratio_.

### Force-velocity slope

**Fig. 2:**
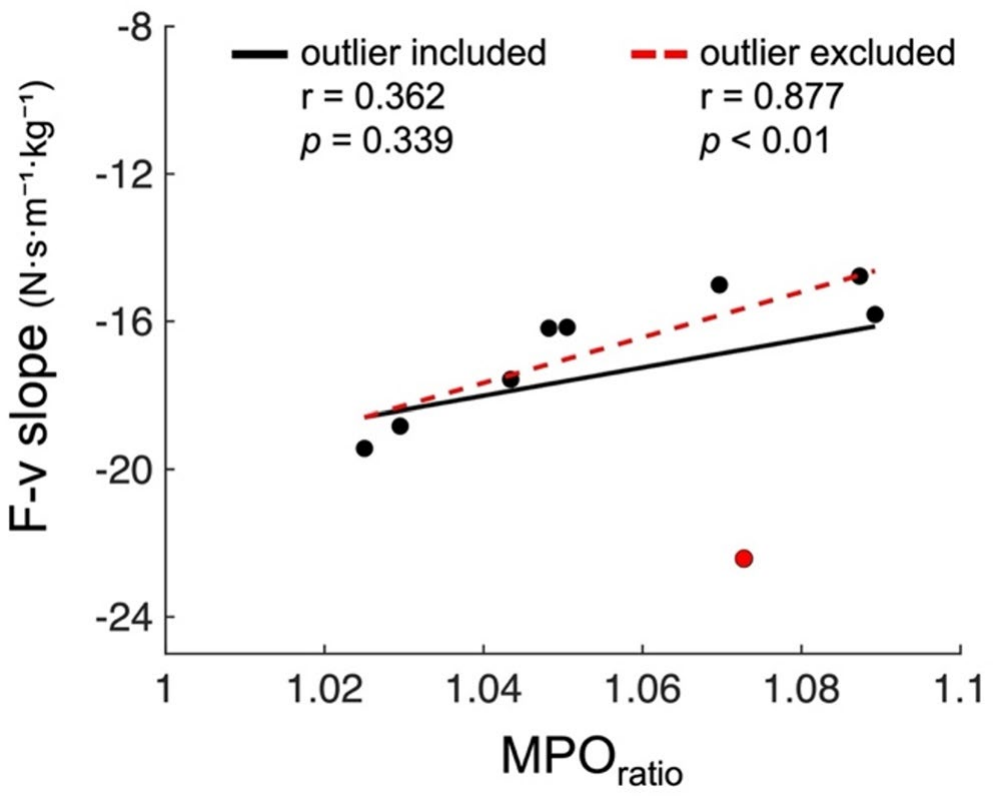
Correlation between the slope of the force-velocity relation (F-v slope, in N·s·m⁻¹·kg⁻¹) and the ratio between 1500 m Mean Power Output and 2000 m Mean Power Output (MPO_ratio_). One participant proved to be an influential datapoint (Cook’s D = 4.455, indicated in red). The solid black line is the best fitting line including this datapoint (95% CI [−0.398, 0.827]), the dashed red line is the best fitting line excluding the datapoint (95% CI [0.452, 0.978])

The high correlation between measured velocity and imposed force (*r* = 0.97–0.99, *p* < 0.01) indicates that F-v relations were linear. F-v slope values normalized to body weight ranged from − 14.77 to −22.42 N·s·m⁻¹·kg⁻¹ with an average of −17.36 (± 2.49) N·s·m⁻¹·kg⁻¹. The correlation between MPO_ratio_ and the F-v slopes, displayed in Fig. 2 (solid line), is moderately positive (*r* = 0.362, r^2^ = 0.131, *p* = 0.339, 95% CI [−0.398, 0.827]). The positive sign of the correlation coefficient indicates that individuals with a more speed-oriented profile (i.e., smaller magnitude of the slope), perform relatively better at 1500 m compared to 2000 m.

Visual inspection of the scatterplot suggests the presence of an influential datapoint. Additional analysis using Cook’s distance (D) showed that this datapoint has a Cook’s D of 4.455, exceeding the commonly accepted threshold (D > 1), thereby confirming it to be a datapoint of high influence (Cook 2000). Excluding this participant increased the correlation coefficient from moderate to very strong (*r* = 0.877, r^2^ = 0.77, *p* = 0.004, 95% CI [0.452, 0.978]), which is visualised in Fig. 2 by the dashed line.

## Discussion

This study related two proxies for athlete phenotype (locomotor profiling and force-velocity profiling) to power output differences between 1500 m and 2000 m rowing ergometer performances. The findings indicate that, among athletes with comparable 2000 m performances, those with a higher power reserve ratio and a lower F-v slope magnitude are likely to excel over their counterparts in 1500 m performances. Both profiling approaches approximate underlying muscle phenotype and thereby lend evidence to the hypothesis that more speed-oriented athletes are better suited to the shortened distance. This can have important implications for athlete training and selection.

The PRR can be established with relative ease, using readily available equipment (rowing ergometer and extracted stroke-for-stroke data). Since these tests are performed on a rowing ergometer, which has similar kinetics to on-water rowing (Lamb 1989), this approach has high ecological validity. We noticed relatively high variability in the execution of the laboratory tests (particularly the 10s_max_-test) regarding stroke rate and length at the front and back end, which likely created a notable level of noise. This likely contributed to the p-value not reaching significance level. It could be beneficial to establish peak power with a fixed stroke rate protocol, similar to the one by Astridge et al. (2025). Since the squat test performance used for F-v profiling is subject to less confounders, a higher internal validity may be assumed for this profiling approach. In our sample, a clear influential datapoint was noted, who’s exclusion led to a remarkable improvement in the correlation (from *r* = 0.362, *p* = 0.339, to *r* = 0.877, *p* = 0.004). This participant visibly struggled with the explosive execution of the squats, as evidenced by the fact that this participant reached the lowest maximal velocity across the whole sample and had the largest F-v slope magnitude.

We conclude that a speed-oriented profile is of increased importance in 1500 m racing. Due to the nature of the Olympics, where a fraction of a second can be decisive for winning a medal, treating 1500 m race preparations (e.g., training and selection) according to customary 2000 m specific protocols could result in suboptimal performance. Athlete phenotype should be shifted slightly toward a speed-oriented profile, especially in endurance phenotype rowers. This may be achieved by increased anaerobic stimuli or plyometric training, which has been shown to increase fascicle length in female rowers (van der Zwaard et al. 2021). Increased fascicle length can lead to higher maximal contractile velocity on the whole-muscle level (Lieber and Fridén 2000), which will likely lead to a smaller F-v slope magnitude. Notwithstanding, aerobic capacity remains highly important. Increases in peak power have been shown to be associated with decreases in endurance performance (i.e., Fyfe et al. 2014), which would likely have adverse effects on rowing performance. We suggest that training ought not to be shifted dramatically but in a subtle and well-planned manner that allows athletes to maintain their aerobic capacity while increasing peak power and muscle shortening velocities. In line with the reported training methodology differences between 800 m - and 1500 m track running (Haugen et al. 2021), we suggest that training volume could be slightly lower with a higher proportion of training at or above anaerobic threshold and supplementary strength, power, and plyometric work.

Another practical application of this study’s findings would be in crew selection. Our research suggests that an athlete with a smaller magnitude F-v slope and higher PRR will perform better in a shorter duration event. It would therefore be appropriate to put the most speed-oriented athletes into the shorter duration larger boats (i.e., 8+, 4x, 4-) and those oriented towards an endurance-profile into smaller boats (i.e., 2x, 2-, 1x). This recommendation pertains to both 1500 m and 2000 m rowing. Rowers with a particularly speed-oriented profile are perhaps best suited to the emerging coastal rowing beach sprint category.

To our knowledge this is the first study that investigates the potential of locomotor profiling and F-v profiling in rowing. We suggest that both approaches could lead to valuable insights in the fields of talent identification and exercise prescription, particularly because a range of athlete physiologies can succeed in medium-distance endurance sports such as rowing. Profiling may allow coaches to allocate their rowers to coastal rowing or the appropriate flat-water rowing boat class depending on their profiles.

### Limitations and future research

The sample size of this study is a clear limitation. This also contributed to a non-significant correlation for the locomotor profile. Despite only testing nine athletes, we were able to detect clear tendencies. These tendencies should be confirmed in a larger sample. Unfortunately, we were unable to recruit enough highly trained female athletes, forcing us to exclude female athletes from the sample. The variability in time intervals between testing sessions (ranging from one hour to two weeks) may have introduced noise. However, pilot testing and post hoc analyses comparing same-day and different-day performances both indicated that these differences in rest time did not influence performance outcomes.

We postulate that our profiling approaches can be valuable tools in athlete selection, with the potential to guide optimal planning of training towards competitive success. Future research should focus on establishing profile benchmarks for different rowing distances and other endurance sports. Research is needed on the trainability towards optimal profiles for different race durations. It has previously been suggested that locomotor profiling could be used to individualize and optimize training stimuli (Sandford et al. 2021), which has yet to be studied in rowing. We envision that optimal training not only depends on the optimal profile required, but also on the current profile of an individual athlete, as well as other personal traits such as training status and age, and that thus an individualistic approach is required.

## Conclusion

This study investigated whether locomotor profiling with the Power Reserve Ratio and force-velocity profiles correlate with better relative performances in the 1500 m rowing distance at the LA28 Olympics compared to standard 2000 m racing. We conclude that rowers with relatively high anaerobic capacities and smaller F-v slope magnitudes fare relatively better at 1500 m and are thus favoured for the shortened distance. These differences point towards a more speed-oriented profile with higher percentage of fast twitch muscle fibres. While rowing remains an aerobically dominant sport, the aforementioned differences should not be ignored. Increasing anaerobic capacity and incorporating speed-specific training methodology could now, more than ever, make a difference.

## Data Availability

The datasets generated during and/or analysed during the current study are available from the corresponding author on reasonable request.

## Abbreviations

10s_max_-test: Ten stroke maximal test
F-v: Force-velocity
GXT: Graded exercise test
LA28: Los Angeles 2028 Olympic Games
MAP: Maximal aerobic power
MPO: Mean power output
MPO_1500_: Mean power output during the 1500 m time trial
MPO_2000_: Mean power output during the 2000 m time trial
MPP: Maximal peak power
PRR: Power Reserve Ratio
RPE: Rating of perceived exertion
TT_1500_: 1500 m ergometer time trial
TT_2000_: 2000 m ergometer time trial
V̇O_2_max: Maximal oxygen uptake per minute
